# A Forgotten Cause of Chronic Dizziness in the Elderly: Exercise‐Induced Vertebrobasilar Insufficiency due to Grade III Subclavian Steal Syndrome

**DOI:** 10.1002/ccr3.73355

**Published:** 2026-08-26

**Authors:** Said Abdirahman Ahmed, Osman Farah Dahir, Abdijalil Abdullahi Ali, Abdishakur Abdiqadir Mahdi, Abdillahi Mohamed Abdi, Ahmed Elmi Abdi, Ahmed Shafie Aden, Ishak Ahmed Abdi, Mohamud Mire Waberi, Mohamed Abdullahi Mohamud, Abdullahi Mohamed Hassan Fujeyra, Mohamed Sheikh Hassan, Feyza Aksu, Mahad Sadik Mukhtar, Nurto Abdulkadir Said, Ismail Gedi Ibrahim

**Affiliations:** ^1^ Department of Cardiology Mogadishu Somali‐Turkish Training and Research Hospital Mogadishu Somalia; ^2^ Faculty of Medicine and Surgery Salaam University Mogadishu Somalia; ^3^ Department of Cardiovascular Surgery Mogadishu Somalia Türkiye Training and Research Hospital Mogadishu Somalia; ^4^ Department of Radiology Mogadishu Somali‐Turkish Training and Research Hospital Mogadishu Somalia; ^5^ Faculty of Medicine & Surgery Somali National University Mogadishu Somalia; ^6^ Department of Neurology Mogadishu Somali‐Turkish Training and Research Hospital Mogadishu Somalia; ^7^ Department of Pulmonology Mogadishu Somali‐Turkish Training and Research Hospital Mogadishu Somalia; ^8^ Department of Public Health Mogadishu University Mogadishu Somalia

**Keywords:** chronic dizziness, duplex ultrasonography, elderly patient, subclavian steal syndrome, vertebral artery flow reversal, vertebrobasilar insufficiency

## Abstract

Chronic dizziness in elderly patients, particularly when reproducibly triggered by upper limb exertion, should prompt evaluation for arm‐exertion‐provoked vertebrobasilar hypoperfusion due to subclavian steal syndrome. Duplex ultrasonography can identify complete retrograde vertebral artery flow and confirm complete Grade III subclavian steal physiology, highlighting a treatable vascular cause that may otherwise be misattributed to vestibular, medication‐related, or neurodegenerative disease.

## 1 Introduction

Chronic dizziness in elderly patients represents a frequent yet diagnostically challenging clinical presentation. Although vestibular disorders, orthostatic hypotension, medication effects, and neurodegenerative disease are common etiologies, vascular causes—particularly vertebrobasilar insufficiency—remain important and often under‐recognized contributors [1]. Subclavian steal syndrome (SSS) is a hemodynamic condition resulting from significant stenosis or occlusion of the proximal subclavian artery, leading to retrograde flow in the ipsilateral vertebral artery and diversion of blood from the posterior cerebral circulation to the upper extremity [2]. When symptomatic, this phenomenon may manifest as dizziness, vertigo, syncope, ataxia, visual disturbances, or upper limb claudication, particularly during arm exertion [3].

Recent reports indicate that SSS continues to be an overlooked cause of chronic dizziness, especially in elderly populations where symptoms are frequently attributed to benign vestibular disorders [4]. The prevalence of subclavian artery stenosis increases with age due to progressive atherosclerosis, and it often coexists with other manifestations of systemic vascular disease [5]. However, only a proportion of patients develop symptomatic vertebrobasilar insufficiency, typically precipitated by increased upper limb demand that accentuates retrograde vertebral flow [6].

Advances in noninvasive vascular imaging have significantly improved diagnostic accuracy. Duplex ultrasonography remains the first‐line modality, enabling characterization of vertebral artery waveform morphology and identification of complete retrograde flow consistent with Grade III steal physiology [7]. Cross‐sectional imaging with CT angiography or MR angiography provides definitive anatomical confirmation of proximal subclavian artery occlusion and supports therapeutic planning [8]. Current literature also emphasizes the importance of bilateral blood pressure measurement and careful vascular examination in patients presenting with unexplained dizziness [9].

With increasing life expectancy and a rising burden of atherosclerotic disease, recognition of SSS as a potentially reversible vascular cause of chronic vertebrobasilar hypoperfusion symptoms is clinically important. This case illustrates a diagnostically challenging presentation of symptomatic Grade III SSS in a nonagenarian. The distinctive value of this case is the reproducible induction of vertebrobasilar hypoperfusion symptoms by left upper limb exertion in a 90‐year‐old patient, with imaging‐confirmed complete Grade III subclavian steal physiology. This combination is clinically important because chronic dizziness in elderly patients is often attributed to vestibular, medication‐related, or neurodegenerative causes, potentially delaying recognition of a treatable vascular condition.

## 2 Case History/Examination

A 90‐year‐old woman with a medical history significant for long‐standing diabetes mellitus and hypertension presented with chronic dizziness of several months' duration. The episodes were described as lightheadedness and imbalance, consistent with vertebrobasilar hypoperfusion symptoms, and were reproducibly precipitated by use of the left upper limb and relieved with rest. There was no history of syncope, focal neurological deficit, chest pain, or palpitations.

On physical examination, cardiovascular and neurological assessments were unremarkable. A significant inter‐arm blood pressure difference was noted. Electrocardiography demonstrated normal sinus rhythm without ischemic changes. Transthoracic echocardiography revealed mild left ventricular hypertrophy with preserved systolic function.

## 3 Methods

### 3.1 Differential Diagnosis

The primary differential diagnoses considered for chronic, exertion‐induced dizziness included: benign paroxysmal positional vertigo (BPPV), orthostatic hypotension, cardiac arrhythmias, cerebrovascular disease (including vertebrobasilar insufficiency), and medication side effects.

### 3.2 Investigations

Given the exertional nature of her symptoms and the unrevealing initial workup, targeted vascular imaging was pursued.

Laboratory Investigations: Routine investigations, including complete blood count, renal function, electrolytes, and glucose profile, were within normal limits.

**FIGURE 1:**
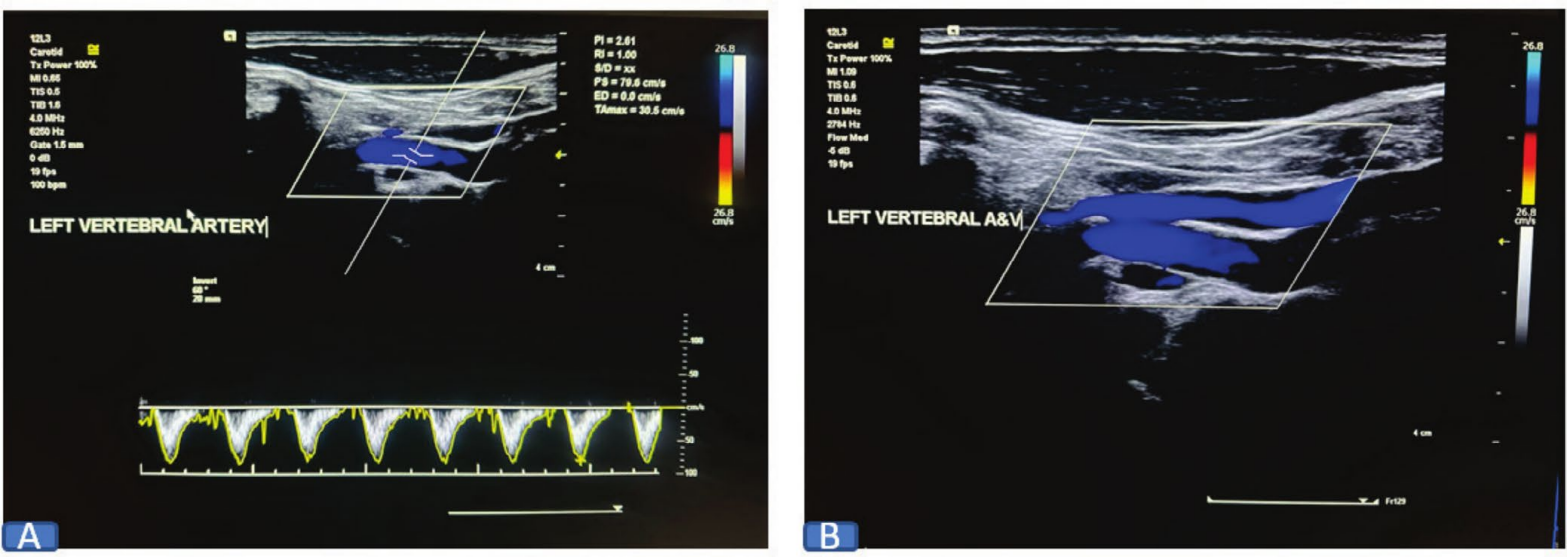
Complete (Grade III) left subclavian steal syndrome secondary to proximal left subclavian artery occlusion.

**FIGURE 2:**
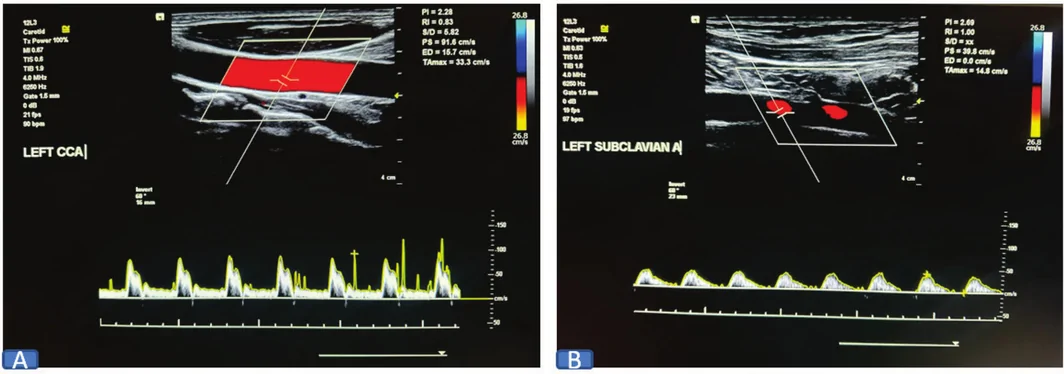
Isolated proximal subclavian artery occlusion and normal left CCA waveform support.

Duplex Doppler Ultrasonography: Evaluation of the left vertebral artery demonstrated complete retrograde vertebral artery flow throughout the cardiac cycle, with the entire spectral waveform located below the baseline, consistent with complete Grade III subclavian steal physiology secondary to symptomatic proximal left subclavian artery occlusion. The color Doppler signal corresponded with the adjacent vertebral vein, supporting reversal of vertebral artery flow direction (Figure 1). Doppler interrogation of the left subclavian artery revealed a low‐velocity monophasic waveform with blunted systolic upstroke and absent diastolic flow, with a peak systolic velocity of approximately 39.8 cm/s and a resistive index of approximately 1.0, indicating complete loss of the normal triphasic morphology (Figure 2A). The left common carotid artery demonstrated a normal antegrade flow pattern, effectively excluding significant ipsilateral carotid inflow disease (Figure 2B).

**FIGURE 3:**
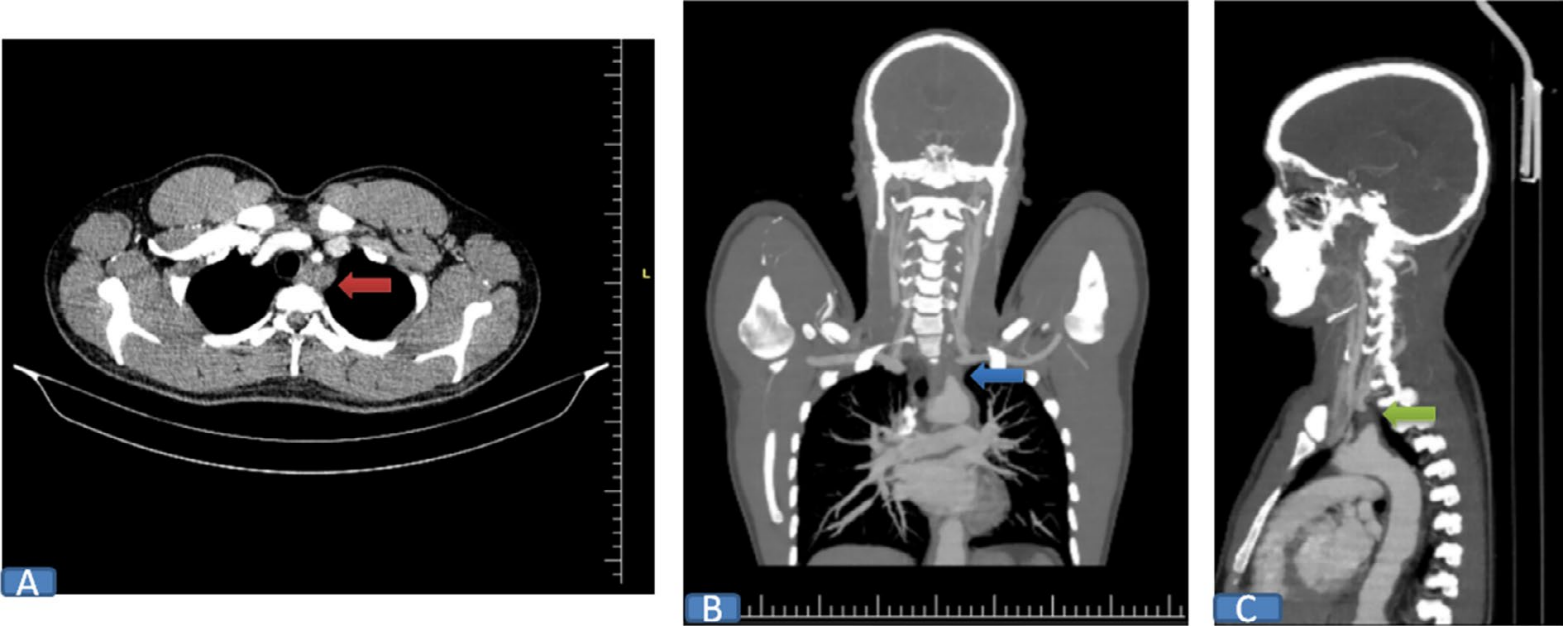
CT angiography demonstrates complete proximal left subclavian artery occlusion, seen as abrupt contrast cutoff on axial image (A, red arrow), coronal image (B, blue arrow), and sagittal image (C, green arrow).

Computed Tomography Angiography: Multiplanar computed tomography angiography confirmed complete proximal left subclavian artery occlusion, demonstrating abrupt contrast cutoff on axial, coronal, and sagittal reconstructions (Figure 3).

### 3.3 Treatment

Based on the clinical presentation and imaging findings, the patient was diagnosed with symptomatic proximal left subclavian artery occlusion causing complete Grade III subclavian steal physiology and arm‐exertion‐provoked vertebrobasilar hypoperfusion symptoms. Endovascular revascularization via percutaneous transluminal angioplasty with possible stenting was recommended because of the reproducible posterior circulation symptoms, complete retrograde vertebral artery flow, and anatomical confirmation of proximal left subclavian artery occlusion.

The anticipated benefit of intervention was restoration of antegrade vertebral artery flow, improvement in vertebrobasilar hypoperfusion symptoms, and improved left upper limb perfusion. However, after discussion of the expected benefits and potential procedural risks, including stroke or transient ischemic attack, embolization, arterial dissection, restenosis, access‐site complications, bleeding, and contrast‐related renal injury, the patient and her family elected to defer intervention because of her advanced age and procedural concerns. She was therefore managed conservatively with optimized medical therapy, including antiplatelet treatment, statin therapy, and strict control of cardiovascular risk factors.

## 4 Conclusion and Results

### 4.1 Outcome and Follow‐Up

At discharge, the patient was clinically stable and was advised to avoid excessive left upper limb exertion, which had been identified as the main trigger for her vertebrobasilar hypoperfusion symptoms. At 4‐week follow‐up, she reported clear subjective improvement in dizziness with activity modification and optimized medical therapy, with no episodes of syncope, focal neurological deficit, or hospital readmission. At 6‐month follow‐up, she remained clinically well, with sustained improvement in symptoms and no reported recurrent posterior circulation events. No repeat Doppler ultrasonography or CT angiography was performed during the available follow‐up period, which represents a limitation of this report.

## 5 Discussion

In this case, the key diagnostic issue was not simply the presence of subclavian steal physiology, but the clear clinical correlation between left upper limb exertion and reproducible vertebrobasilar hypoperfusion symptoms. Although subclavian steal may remain asymptomatic, symptom provocation during ipsilateral arm use should raise suspicion for clinically significant steal physiology, particularly in elderly patients with vascular risk factors [10]. The prevalence of subclavian artery stenosis increases with advancing age due to progressive atherosclerosis, and it is commonly associated with traditional cardiovascular risk factors such as diabetes mellitus, hypertension, and dyslipidemia [11]. In elderly individuals, diffuse vascular disease and impaired cerebrovascular reserve may predispose to symptomatic vertebrobasilar insufficiency even in the absence of critical bilateral carotid disease [12]. Our patient's advanced age and vascular comorbidities likely contributed to reduced compensatory capacity within the posterior circulation.

Clinically, symptomatic SSS may present with posterior circulation symptoms, including dizziness, vertigo, presyncope, ataxia, visual disturbance, or syncope, often precipitated by upper limb exertion [13]. Arm claudication or fatigue may coexist but may be absent or underreported in elderly patients with limited baseline activity [14]. In this context, reproducibility of posterior circulation symptoms with ipsilateral arm use, together with an inter‐arm blood pressure difference, should prompt targeted vascular assessment rather than attribution of symptoms solely to vestibular or neurodegenerative causes [15].

Duplex ultrasonography is the first‐line diagnostic modality for suspected SSS and allows hemodynamic grading of steal physiology [16]. Complete retrograde vertebral artery flow throughout the cardiac cycle, as demonstrated in this case, is consistent with complete Grade III subclavian steal physiology and indicates hemodynamically significant proximal obstruction [17]. Preservation of normal antegrade flow in the ipsilateral common carotid artery, as observed in our patient, helps exclude significant carotid inflow disease and supports isolated subclavian pathology [18]. Cross‐sectional imaging with computed tomography angiography provides definitive anatomical confirmation and assists in procedural planning by delineating lesion location, extent, and collateral circulation [19].

This case also reinforces the practical value of bilateral arm blood pressure measurement and symptom‐provocation history, as these simple bedside clues can direct clinicians toward early duplex ultrasonography in patients with otherwise unexplained chronic dizziness.

Management strategies for SSS should be individualized according to symptom burden, hemodynamic severity, anatomical feasibility, comorbidity profile, and patient preference [17]. Symptomatic subclavian artery stenosis or occlusion causing vertebrobasilar hypoperfusion symptoms can be considered for revascularization, particularly when symptoms are reproducible, functionally limiting, or associated with posterior circulation ischemia [17]. Contemporary European guideline‐based practice also supports individualized management of peripheral arterial disease, with attention to symptom severity, vascular territory, procedural risk, and cardiovascular risk reduction [20]. In anatomically suitable proximal subclavian lesions, endovascular revascularization with percutaneous transluminal angioplasty and stenting is commonly favored because it is less invasive than open surgical repair and has demonstrated high technical success and favorable patency outcomes in contemporary series [19].

However, conservative management remains appropriate in patients with mild or manageable symptoms, those who decline intervention, or those in whom procedural risk is considered to outweigh the expected clinical benefit [17]. Best medical therapy should include antiplatelet treatment, lipid‐lowering therapy, blood pressure control, glycemic control, smoking cessation where relevant, and broader cardiovascular risk‐factor optimization, Because subclavian artery stenosis is usually a manifestation of systemic atherosclerotic disease [20]. In very elderly or frail patients, shared decision‐making is particularly important, balancing the potential benefits of restoring antegrade vertebral artery flow and improving vertebrobasilar hypoperfusion symptoms against procedural risks [17]. These risks include stroke or transient ischemic attack, embolization, arterial dissection, restenosis, access‐site complications, bleeding, and contrast‐related renal injury [19].

In the present case, endovascular revascularization was recommended because the patient had symptomatic proximal left subclavian artery occlusion, complete retrograde left vertebral artery flow, and reproducible left upper limb exertion‐induced vertebrobasilar hypoperfusion symptoms [17]. Nevertheless, after discussion of the expected benefits and procedural risks, the patient and her family elected to defer intervention because of her advanced age and procedural concerns. Conservative management with antiplatelet therapy, statin therapy, cardiovascular risk factor optimization, and activity modification was therefore considered a reasonable patient‐centered approach [20].

This case highlights several important clinical considerations. First, SSS remains a potentially overlooked vascular cause of chronic vertebrobasilar hypoperfusion symptoms in elderly patients. Second, reproducible arm‐exertion‐provoked posterior circulation symptoms should prompt careful vascular examination, including bilateral arm blood pressure measurement. Third, duplex ultrasonography can rapidly confirm complete retrograde vertebral artery flow and complete Grade III subclavian steal physiology, while CT angiography provides anatomical confirmation for treatment planning. Finally, management decisions in nonagenarians require multidisciplinary assessment and shared decision‐making, integrating hemodynamic severity, procedural risk, comorbidity burden, life expectancy, and patient preference.

### 5.1 Limitations

This report has several limitations. First, as a single case report, the findings cannot be generalized to all elderly patients presenting with chronic dizziness or subclavian steal physiology. Second, although the patient demonstrated clinical improvement at 4‐week and 6‐month follow‐up, follow‐up assessment was based primarily on subjective symptom reporting and clinical review. No repeat Doppler ultrasonography or CT angiography was performed to objectively confirm persistent or improved vertebral artery flow after conservative management. Third, because endovascular intervention was deferred, this case cannot provide procedural outcome data or post‐revascularization hemodynamic assessment. Nevertheless, the case remains clinically relevant because it highlights the diagnostic importance of symptom provocation with upper limb exertion, inter‐arm blood pressure difference, and early duplex ultrasonography in elderly patients with otherwise unexplained chronic dizziness.

## Author Contributions

**Said Abdirahman Ahmed:** conceptualization, resources. **Osman Farah Dahir:** conceptualization, formal analysis. **Abdijalil Abdullahi Ali:** data curation, formal analysis. **Abdishakur Abdiqadir Mahdi:** methodology, resources. **Abdillahi Mohamed Abdi:** investigation, methodology. **Ahmed Elmi Abdi:** data curation, formal analysis. **Ahmed Shafie Aden:** investigation, writing – original draft, writing – review and editing. **Ishak Ahmed Abdi:** conceptualization, resources. **Mohamud Mire Waberi:** project administration, writing – original draft, writing – review and editing. **Mohamed Abdullahi Mohamud:** data curation, formal analysis. **Abdullahi Mohamed Hassan Fujeyra:** investigation, resources, software. **Mohamed Sheikh Hassan:** conceptualization, data curation. **Feyza Aksu:** conceptualization, data curation. **Mahad Sadik Mukhtar:** data curation, supervision. **Nurto Abdulkadir Said:** conceptualization, visualization. **Ismail Gedi Ibrahim:** investigation, methodology.

## Funding

The authors have nothing to report.

## Disclosure

Permission to Reproduce Material: All figures and images are original and have not been previously published.

## Ethics Statement

The authors have nothing to report.

## Consent

Written informed consent for publication of this case report and accompanying images was obtained from the patient's family.

## Conflicts of Interest

The authors declare no conflicts of interest.

## Data Availability

All relevant data are included within the article. Further information is available from the corresponding author upon reasonable request, subject to patient confidentiality.
